# Annotations

**Published:** 1893-02-11

**Authors:** 


### Annotations.

An outbreak of cholera is anticipated in the coming spring. The importance of anticholeraic treatment is therefore obvious. But, besides, inoculation against a variety of diseases is the chief hope of medical scientists, as well as their most advanced line of progress, at the present time. The critical portion of the medical profession should lend its mind to the full and adequate consideration of this burning question. It is essential that new facts be gathered and collated with all that have gone before, and that the best mental power which the profession has should set itself steadily and perseveringly to work. Will inoculation justify the hopes of inoculators? Are we really at the beginning of a new era in preventive medicine—in preventive medicine on the most extensive scale? The prospect is dazzling in its hopefulness and its brilliancy. At Netley Hospital, the professor of pathology, Dr. A. E. Wright, and the assistant-professor, Surgeon-Captain D. Bruce, invited M. Haffkine to demonstrate his methods to the assembled students, and have published interesting and philosophical expositions of the demonstration. Microbial infections, say the experimenters, may be separated into two classes: those infections which produce septicæmia, and those which produce intoxication. This division, though convenient in sense, is not logical in form, and will have to be amended: for what is "septicæmia" but "blood-poisoning," and what is "intoxication" but "poisoning of the blood"? However, that point need not be laboured in a brief notice like the present. The facts of immediate importance are that two series of guinea pigs were experimented upon, and with very striking results. In brief, the first series were inoculated with prophylactic virus, and afterwards injected with cholera virus of differing strength, with the result that two out of the three guinea pigs survived the injection of very strong virus, and became quite well in a day or two after it. The third succumbed to a very powerful injection. On the other hand, the second series, none of which had been inoculated with the preventive virus, all died, and one of them succumbed to a virus of very greatly diminished strength. The two prepared rabbits survived injections of the strength of 1-8th and 1-6th. The three rabbits which had not been previously inoculated died after being injected with tubes of a strength of 1-8th, 1-16th, and 1-32nd. These are significant facts to those who can estimate them at their true value. M. Haffkine has already treated about fifty human subjects with protective virus. The treatment has not in any case produced alarming consequences, and it remains to be seen what amount of protection has been obtained against cholera in its natural and epidemic form.

*Experimental anticholeraic inoculations.*

It rejoices the heart of the medical enthusiast to find that the merchant sailors of our country and the world's seas are to profit markedly by the application of physiological science to practice. All readers of newspapers are aware that a victualling committee, appointed by the shipowners themselves, has been considering the question of "Jack's" food for a long time. The committee has worked thoroughly and honestly, and has recommended changes of real value. Inquiry has proved, not that sailors have been badly used, either intentionally or otherwise, but that they have not been used scientifically well. In other words, science points out various improvements, which ought to be effected in the feeding of merchant sailors, and those improvements, which are to be immediately carried out, are such as will greatly increase the comfort, as well as improve the health of seamen. The fact of chief importance is that an attempt is to be made to substitute preserved meat for salt, and fresh vegetables for preserved, on as large a scale as is practicable. In port, neither salt nor preserved meat is to be the rule, but meat that is freshly killed, and, of course, entirely fresh vegetables. Next in importance to the question of food supply is that of cooking. Sea-cooks are to be taught to cook. If this can be done; if to every merchant vessel there be appointed a man who can and will cook well, a new era of health and comfort is about to dawn for sailors. Carlyle railed at "cookery" as one of the gods of modern idolatry. If he had been wiser he would have exalted cookery as one of the most fundamentally important of modern arts. The sailor especially, with his long voyages, his stern battlings against wind and weather, his dangers by day and night, needs to be kept in heart and health by wholesome, satisfying, and comfortable meals. It has always been said that the English soldier's stomach is the perennial source of his courage. It must be so to a large extent with sailors. With good digestion and plenty of appetising food Carlyle has more wisely said a "a man may front much." We may hope that with the substitution of fresh and preserved meats for salt, and of fresh vegetables for preserved, and with good cooking, the bad old days of scurvy and general ill-health are for ever past. The *British Medical Journal*, Dr. Spooner, the Medical Officer of the Board of Trade in Liverpool, and Dr. Sedgewick Saunders, of London, are alike to be congratulated on the successful termination of an arduous, prolonged, and important piece of public work.

*The Food of "Poor Jack."*

Last week, after four days' illness, died Mr. J. H. Hewer of Highbury New Park, one of the most trusted practitioners, and one of the most esteemed men in the north of London. Mr. Hewer had been in practice more than forty years. It was often said of him that "London was his parish." His peculiarity was that once he had gained a patient he never lost him. If the patient removed to Hampstead, or to Kensington, or to Brixton, or even further away from Highbury, he always insisted upon sending for Mr. Hewer when he was ill. Indeed, it was perhaps to just such a distant call that this exemplary surgeon owed his untimely death. For though Mr. Hewer could hardly have been much less than sixty-five, he seemed so hale and hearty that one would have guaranteed him twenty years more of life and fifteen of practice. But last week, in cold, damp weather, he was summoned at midnight to a patient in a distant southern suburb ten or twelve miles away from his own house. Soon afterwards he developed pneumonia, and by a pathetic coincidence, he and his distant patient died on the same day. One hardly knows what to say of a man like Mr. Hewer, which will not appear exaggerated to those who did not know him. He was an admirable practitioner, full of experienced resource, calm, courageous, and eminently kind and gentle. His mere presence in the house seemed to bring hope and strength and the beginning of restoration. His older patients—and their name was "legion"—will mourn his loss with peculiar sorrow. They had reckoned upon having him in their declining years as a sure comforter and protector against premature death. Mr. Hewer died in harness. He never expected to do otherwise. His life was an example and a warning. He had one of the largest practices in Great Britain, and an exemplary and economical family; and yet, as he himself once said to the present writer, he had "always had his nose to the grindstone, and expected to have it always there to the end of his life." Forty years of such professional work as Mr. Hewer's would have achieved fortune in any other profession than that of medicine.

